# Soluble TNF Receptors Are Associated with Infarct Size and Ventricular Dysfunction in ST-Elevation Myocardial Infarction

**DOI:** 10.1371/journal.pone.0055477

**Published:** 2013-02-06

**Authors:** Lennart Nilsson, Aleksander Szymanowski, Eva Swahn, Lena Jonasson

**Affiliations:** 1 Division of Cardiology, Department of Medical and Health Sciences, Linköping University, Linköping, Sweden; 2 Department of Cardiology, University Hospital, Linköping, Sweden; The University of Texas Health Science Center, United States of America

## Abstract

**Objectives:**

The aim of the study was to investigate circulating markers of apoptosis in relation to infarct size, left ventricular dysfunction and remodeling in an ST-elevation myocardial infarction (STEMI) population undergoing primary percutaneous coronary intervention (PCI).

**Background:**

Immediate re-opening of the acutely occluded infarct-related artery via primary PCI is the treatment of choice in STEMI to limit ischemia injury. However, the sudden re-initiation of blood flow can lead to a local acute inflammatory response with further endothelial and myocardial damage, so-called reperfusion injury. Apoptosis is suggested to be a key event in ischemia-reperfusion injury, resulting in LV-dysfunction, remodeling and heart failure.

**Methods:**

The present study is a prespecified substudy of the F.I.R.E. trial. We included 48 patients with STEMI undergoing primary PCI. Blood samples were collected prior to PCI and after 24 hours. Plasma was separated for later analysis of soluble tumor necrosis factor receptor (sTNFR) 1, sTNFR2, sFas and sFas ligand (sFasL) by ELISA. Infarct size, left ventricular (LV) dysfunction and remodeling were assessed by cardiac magnetic resonance imaging at five days and four months after STEMI.

**Results:**

The levels of sTNFR1 at 24 h as well as the relative increases in sTNFR1 and sTNFR2 over 24 h showed consistent and significant correlations with infarct size and LV-dysfunction at four months. Moreover, both sTNFRs correlated strongly with Troponin I and matrix metalloproteinase (MMP)-2 measurements. Soluble Fas and sFasL did not overall correlate with measures of infarct size or LV-dysfunction. None of the apoptosis markers correlated significantly with measures of remodeling.

**Conclusions:**

In STEMI patients, circulating levels of sTNFR1 and sTNFR2 are associated with infarct size and LV dysfunction. This provides further evidence for the role of apoptosis in ischemia-reperfusion injury.

## Introduction

Coronary artery disease (CAD) is the leading cause of death in the western world. In many patients the first clinical presentation of CAD is an acute myocardial infarction. Immediate re-opening of the acutely occluded infarct-related artery via primary percutaneous coronary intervention (PCI) is the treatment of choice to limit ischemic injury in the setting of ST-elevation myocardial infarction (STEMI) [1], [2]. However, the sudden re-initiation of blood flow achieved with primary PCI can give rise to further endothelial and myocardial damage, so-called reperfusion injury [3], [4].

Ischemia and reperfusion associated myocardial injury (IR-injury) involves a wide range of pathological processes. Vascular leakage, activation of cell death programs, transcriptional reprogramming, no reflow phenomenon and innate and adaptive immune activation all contribute to tissue damage, thereby determining the infarct size [5]. Cell death programs include apoptosis, necrosis and autophagy associated cell death. Apoptosis has been implicated in each step ranging from myocardial IR- injury, to left ventricular dysfunction and remodelling, and to the development of end-stage heart failure [6]–[9]. In a recent study from our group, we found that early elevations of matrix metalloproteinase (MMP)-2 in plasma correlated strongly with infarct size and left ventricular dysfunction in a STEMI population, indicating that MMP-2 might play an important role in IR-injury [10]. Indeed, experimental studies and animal models have demonstrated various mechanisms, including apoptosis, by which MMP-2 activation can mediate IR-injury [11].

There are two major pathways for apoptosis: the intrinsic mitochondrial pathway and the extrinsic pathway, involving the binding of soluble or cell membrane-bound ligands to cell surface receptors [12]. Feedback loops aiming at limiting the apoptotic rate include down regulation of the transmembrane receptor and shedding of its extracellular part, which through binding to circulating ligands can limit ligand-transmembrane receptor interaction [13]. Among these circulating markers of apoptosis, soluble Fas (sFas), and soluble tumor necrosis factor receptor 1 (sTNFR1) are elevated in patients with acute myocardial infarction [14]–[16], with some clinical evidence of the latter being both a predictor of the development of heart failure and of overall mortality [15], [16].

This study is a pre-specified substudy of the F.I.R.E. trial, which studied the effects of the fibrin –derived peptide Bβ_15–42_ (FX06) on reperfusion injury in patients undergoing primary PCI for STEMI [17], [18]. Infiltration and activation of leukocytes is an important event in myocardial reperfusion injury [5], [19]–[21]. FX06 is a naturally occurring fragment of fibrin, which binds to the VE-cadherin receptor on endothelial cells, thereby inhibiting leukocyte transmigration through gap junctions and tissue inflammation injury [22], [23]. The positive effects of FX06 on the inflammatory response are demonstrated by a decrease in cytokines and interleukins following coronary occlusion and reperfusion in pigs [24]. In occlusion-reperfusion studies in rats, administration of FX06 caused a significant 40% reduction in infarct size compared to placebo [23].

Cardiac magnetic resonance imaging (CMR) has consistently been found to accurately determine the size of myocardial infarct as well as left ventricular volumes used to calculate measures of left ventricular dysfunction and remodelling [25]. They all reflect the extent of myocardial ischemia-reperfusion injury and are strongly associated with poor prognosis [26].

The aim of this study was to analyze the circulating levels of apoptosis markers, sTNFR1, sTNFR2, sFas and sFasL, in STEMI patients prior to and 24 hours after primary PCI. Furthermore, we wanted to investigate potential correlations between these biomarkers and measures of infarct size, left ventricular dysfunction and remodelling as assessed by CMR. Lastly, we aimed to study the effect of FX-06 on soluble markers of apoptosis.

## Methods

### Study Population

This study is a pre-specified substudy of the F.I.R.E. trial. The study design and results of the F.I.R.E. trial (http://clinicaltrials.gov, NCT00326976) have been published elsewhere [17], [18]. In brief, the F.I.R.E. trial was a double-blind, randomized, placebo-controlled multicentre-trial performed in 2006–2008, including 234 patients, to study the effects of FX06 on reperfusion damage in patients undergoing primary PCI for STEMI presenting within 6 hours from onset of symptoms. No treatment differences attributable to FX06 were observed in primary or secondary outcome measures [18]. The present substudy was designed by three of the authors (LN, LJ, ES). Complete sample sets were obtained from 46 subjects at 10 of the participating sites. Aside from study medication, patients were treated according to current ESC STEMI guidelines [27]. The study subjects received aspirin (n = 45, 98%), clopidogrel (n = 46, 100%), betablockers (n = 46, 100%) and glycoprotein IIb/IIIa-inhibitors (n = 36, 78%).

### Ethical Considerations

The study protocol was approved by all local ethic committees of the participating sites and written informed consent was obtained from all patients.

### CMR Imaging

The CMR protocol has previously been described in detail [18]. All CMR studies were analyzed at the central MR Evaluation Centre, University Hospital Basel (Basel, Switzerland) by a single experienced CMR reader who was blinded to study groups followed by a blinded review by a level III CMR expert. Intraobserver variability was assessed for the primary reader in a subset of 40 randomly chosen studies and the intraclass correlation was 0.85 for 26 studies from day 5.

### Biochemical Analysis

Blood samples for analysis of sTNFR1, sTNFR2, sFAS, sFASL, MMPs, tissue inhibitors of metalloproteinases (TIMPs) and myeloperoxidase (MPO) were collected in vacutainer tubes (using sodium heparin as anticoagulant) at baseline immediately before primary PCI and at 24 hours. Samples were centrifuged within 30 minutes to separate plasma, which then was stored immediately at −20°C and within a week at −70°C until analyzed. Soluble TNFR1, sTNFR2, sFAS and sFASL were analyzed using commercially available ELISA kit (R&D Systems Europe, Abingdon, United Kingdom). The lower limits of detection were 0.8 pg/mL (sTNFR1), 0.6 pg/mL (sTNFR2), <20 pg/mL (sFAS), and 2.7 pg/mL (sFASL), respectively. The interassay CVs ranged from 0.6–5.1% for sTNFR 1, 5.7–10.4% for sTNFR2, 2.4–4.5% for sFAS, and 1.5–2.4% for sFASL. Plasma levels of MMPs, TIMPs and MPO were analyzed as previously described [10]. Blood samples for Troponin I (cTnI) were obtained at 24 and 48 hours after admission to hospital. All samples were analyzed in a blinded core laboratory (Spranger Laboratories, Ingolstadt, Germany). cTnI was measured on the Abbott AxSym System (Abbott Diagnostics, Abbott Park, Ill, US) using the second-generation AxSYM Troponin-I ADV assay. The lower limit of detection of the assay is 0.020 ng/ml with a 10% CV value of 0.16 ng/ml.

### Statistical Methods

Based on previous data, we calculated that a sample size of 21 subjects in each treatment group (42 subjects in total) would be sufficient to give 80% power to detect an estimated 20% change in plasma levels of the biomarkers [10]. Baseline and procedural variables are presented as mean with standard deviation or median with interquartile range for non-Gaussian distributed data. Biochemical parameters are all presented as median with interquartile range. Spearman correlations were used to assess associations between variables. Wilcoxon signed ranks test was used to evaluate time-dependent changes in biochemical parameters. Two-tailed P-values <0.05 were considered as statistically significant. No formal statistical tests were used to address the issue of multiple comparisons. However, given the large number of tests performed, thoughtful consideration of the specific context, the pre-test probability and the biological plausibility of each statistically significant association was an important issue. All statistical analyses were performed using SPSS version 17 (SPSS Inc., Chicago, USA).

## Results

We included 46 patients of whom 24 received placebo and 22 FX06 treatment. There were no differences between the FX06 treated group and the placebo group in the levels of any of the biomarkers at any time point. Thus, for further analysis both treatment groups were analyzed as one group. Baseline characteristics of the 46 study participants are summarized in table 1. Of note, mean age of the study group was 61 years and about one third was women. Time from symptom to PCI was approximately two and a half hours, and prior to PCI 98% had TIMI 0 flow, whereas after primary PCI 100% had TIMI 2–3 flow. The median infarct size was 35 gram and 23 gram at five days and four months, respectively.

**Table 1:** Baseline, procedural and outcome measures of the study population (n = 46).

**Baseline variables**	
Age, years (mean, SD)	61 (11)
Female, % (n)	35 (16)
Body-mass index, kg/m^2^ (mean, SD)	27 (3.5)
Anterior infarct location, % (n)	46 (21)
Time-to-PCI, minutes (median, 25^th^, 75^th^ percentile)	152 (115, 280)
**Procedural variables**	
TIMI pre-procedural, % (n)	
0	98 (45)
1	2 (1)
TIMI after PCI, % (n)	
2	6 (3)
3	94 (43)
**Medications**	
Glycoprotein IIb/IIIa, % (n)	78 (36)
Aspirin, % (n)	98 (45)
Betablockers, % (n)	100 (46)
Clopdiogrel, % (n)	100 (46)
**Outcomes**	
Infarct size at 5 days, g (median, 25^th^, 75^th^ percentile)	35 (14, 53)
Infarct size at 4 months, g (median, 25^th^, 75^th^ percentile)	23 (6.0, 38)
LVEF at 5 days, % (median, 25^th^, 75^th^ percentile)	45 (40, 52)
LVEF at 4 months, % (median, 25^th^, 75^th^ percentile)	50 (45, 57)
EDVI at 5 days, ml^3^ (median, 25^th^, 75^th^ percentile)	79 (64, 87)
EDVI at 4 monhts, ml^3^ (median, 25^th^, 75^th^ percentile)	79 (63, 90)
ESVI at 5 days, ml^3^ (median, 25^th^, 75^th^ percentile)	44 (31, 50)
ESVI at 4 months, ml^3^ (median, 25^th^, 75^th^ percentile)	38 (28, 48)

SD, standard deviation; PCI, percutaneous coronary intervention; TIMI, Thrombolysis In Myocardial Infarction; LVEF, left-ventricular ejection fraction; EDVI, end-diastolic volume index; ESVI, end-systolic volume index.

### Plasma Levels of sTNFR1, sTNFR2, sFAS, sFASL, MMPs, TIMPs and MPO During the First 24 hours in STEMI Patients Undergoing Primary PCI

Plasma levels of sTNFR1, sTNFR2, sFAS, sFASL, MMP-2, MMP-8, MMP-9, TIMP-1, TIMP-2 and MPO at 0 and 24 hours, as well as the change in plasma levels over time, are summarized in table 2. Of note, TNFR1, TNFR 2 and FAS all increased significantly from baseline to 24 hours, whereas FAS ligand decreased over time. The data on MMPs, TIMPs and MPO have been published elsewhere [10]. Plasma levels of soluble markers of apoptosis were also measured in an age-matched control group (n = 32). The median (25^th^ percentile, 75^th^ percentile) levels were 1280 pg/mL (1162, 1448) for sTNFR1, 2160 pg/mL (1921, 2258) for sTNFR2, 6758 pg/mL (6273, 7077) for sFAS, and 46.7 pg/mL (43.0, 50.8) for sFASL, respectively. These levels were very similar to and did not differ significantly from the plasma levels of STEMI patients at 0 hours (data not shown).

**Table 2:** Plasma levels of sTNFR1, sTNFR2, sFAS, sFASL, MMPs, TIMPs and MPO during the first 24 hours after STEMI and reperfusion treatment (n = 46).

	0 h	24 h	change (%)	p-value
sTNFR1 (pg/mL)	1256 (1039, 1481)	1617 (1216, 1890)	26 (9.0, 40)	p<0.001
sTNFR2 (pg/mL)	2170 (1787, 2570)	2451 (1982, 3269)	14 (−0.4, 24)	P<0.001
sFAS (pg/mL)	6527 (5375, 8093)	7685 (6146, 8655)	9.9 (−2.7, 27)	P<0.001
sFASL (pg/mL)	45.3 (35.8, 55.0)	43.6 (35.7, 51.8)	−5.3 (−12, 2.7)	P = 0.029
MMP-2 (ng/mL)	196 (172, 222)	191 (161, 216)	−5 (−14, 4)	P = 0.193
MMP-8 (ng/mL)	4.0 (2.2, 5.8)	3.9 (1.8, 6.9)	−6 (−60, 102)	P = 0.808
MMP-9 (ng/mL)	89 (43, 135)	62 (31, 153)	−35 (−65, 45)	P = 0.121
TIMP-1 (ng/mL)	75 (70, 88)	98 (84, 128)	29 (16, 52)	P<0.001
TIMP-2 (ng/mL)	67 (61, 74)	64 (56, 72)	−4 (−15, 3)	P = 0.024
MPO (ng/mL)	1245 (260, 1628)	428 (198, 727)	−65 (−82, −31)	P<0.001

Values are given as median (25^th^, 75^th^ percentile). P-values are shown for Wilcoxon signed ranks test. sTNFR1, soluble tumour necrosis factor receptor 1; sTNFR2, soluble tumour necrosis factor receptor 2; sFAS, soluble FAS; sFASL, soluble FAS ligand; MMP-2, matrix metalloproteinase-2; TIMP, tissue inhibitor of metalloproteinase; MPO, myeloperoxidase; STEMI, ST-elevation myocardial infarction.

### Correlations between sTNFR1, sTNFR2, sFAS and sFASL and Measures of Infarct Size

The infarct size, defined as the late gadolinium enhancement zone (LGE zone) on CMR examination, was assessed at five days and four months after STEMI and primary PCI. All study participants were included in the day five examination, whereas 9 of the 46 patients did not complete the MRI at four months. Correlations between sTNFR1, sTNFR2, sFAS, sFASL and LGE zone are summarized in table 3. Soluble TNFR1 at baseline did not correlate with infarct size, whereas sTNFR1 at 24 hours tended to correlate with infarct size at 5 days and showed a significant positive correlation with infarct size measured at 4 months. Also, the relative change in sTNFR1 levels from 0 to 24 hours correlated significantly with infarct size at 5 days and 4 months. Similar but weaker correlations were seen for sTNFR2. Both sFAS and sFASL at 0 and 24 hours showed a tendency towards a negative correlation with infarct size, especially infarct size at 5 days, but none of those correlations were statistically significant.

**Table 3:** Correlations between sTNFR1, sTNFR2, sFAS and sFASL and measures of infarct size.

		Total LGEat 5 days	Total LGEat 4 months
sTNFR1	0 h	−0.014	0.037
	24 h	0.231	0.358*
	% change	0.412*	0.385*
sTNFR2	0 h	0.005	0.034
	24 h	0.138	0.203
	% change	0.356*	0.369*
sFAS	0 h	−0.150	−0.039
	24 h	−0.304	−0.099
	% change	−0.242	−0.097
sFASL	0 h	−0.236	−0.192
	24 h	−0.210	−0.174
	% change	0.036	−0.021

Values are given as rho-values from Spearman test.

* p<0.05.

sTNFR1, soluble tumour necrosis factor receptor 1; sTNFR2, soluble tumour necrosis factor receptor 2; sFAS, soluble FAS; sFASL, soluble FAS ligand; LGE, late gadolinium enhancement zone.

### Correlations between sTNFR1, sTNFR2, sFAS, sFASL and Measures of Left Ventricular Dysfunction and Remodeling

Findings are summarized in table 4. Of interest, sTNFR1 at 24 hours as well as the relative change in sTNFR1 from 0 to 24 hours, showed a tendency towards a negative correlation with LVEF at 5 days and a significant negative correlation at 4 months. Also, sTNFR2 at 24 hours showed a trend of a negative correlation with LVEF at 4 months. We found similar tendencies for soluble FAS and FAS ligand, however, none of those were significant. For dEDVI and dESVI, we found no consistent correlations with any of the biomarkers.

**Table 4:** Correlations between sTNFR1, sTNFR2, sFAS and sFASL and measures of left ventricular dysfunction and remodeling.

		LVEF% at5 days	LVEF% at4 months	dEDVI	dESVI
sTNFR1	0 h	−0.048	−0.177	−0.070	0.097
	24 h	−0.200	−0.354*	−0.237	−0.067
	% change	−0.274	−0.375*	−0.285	−0.253
sTNFR2	0 h	−0.017	−0.155	−0.119	0.071
	24 h	−0.078	−0.253	−0.160	−0.014
	% change	−0.125	−0.192	−0.045	−0.159
sFAS	0 h	−0.022	−0.075	0.061	0.210
	24 h	−0.264	−0.258	0.171	0.196
	% change	0.048	−0.078	0.173	−0.005
sFASL	0 h	−0.257	−0.228	0.035	0.175
	24 h	−0.249	−0.168	−0.106	0.057
	% change	−0.016	0.087	−0.202	−0.194

Values are given as rho-values from Spearman test.

* p<0.05.

sTNFR1, soluble tumour necrosis factor receptor 1; sTNFR2, soluble tumour necrosis factor receptor 2; sFAS, soluble FAS; sFASL, soluble FAS ligand; LVEF, left ventricular ejection fraction; dEDVI, change in end-diastolic volume index from 5 days to 4 months; dESVI, change in end-systolic volume index from 5 days to 4 months.

### Correlations between sTNFR1, sTNFR2, sFAS, sFASL and Troponin I, MMPs, TIMPs and MPO

Correlations with Troponin I and MMP-2 are summarized in table 5. We found significant positive correlations between sTNFR1 and sTNFR2 and Troponin I at 24 hour. Circulating levels of FAS and FAS ligand did not show any consistent correlations with Troponin I. Soluble TNFR1, sTNFR2 and sFAS all showed positive correlations with MMP-2, whereas FAS ligand was negatively correlated with MMP-2 at both 0 and 24 hours. MMP-8, MMP-9, TIMP-1, TIMP-2 and MPO did not correlate significantly with any of the soluble markers of apoptosis (data not shown). Data on Troponin I, MMPs, TIMPs and MPO measurements from this substudy population have been published previously [10].

**Table 5:** Correlations between sTNFR1, sTNFR2, sFAS, sFASL and Troponin I and MMP-2.

		Troponin I at 24 hours	MMP-2 at 24 hours
sTNFR1	0 h	0.101	0.397**
	24 h	0.615**	0.595**
	% change	0.613**	0.478**
sTNFR2	0 h	0.072	0.311*
	24 h	0.553**	0.595**
	% change	0.550**	0.493**
sFAS	0 h	−0.125	0.182
	24 h	0.136	0.582**
	% change	0.368*	0.481**
sFASL	0 h	−0.103	−0.458**
	24 h	−0.064	−0.387**
	% change	0.046	0.194

Values are given as rho-values from Spearman test.

* p<0.05,

** p<0.01.

sTNFR1, soluble tumour necrosis factor receptor 1; sTNFR2, soluble tumour necrosis factor receptor 2; sFAS, soluble FAS; sFASL, soluble FAS ligand; MMP; matrix metalloproteinase.

## Discussion

The main finding of the study was the consistent and significant correlation between 24 hour levels of plasma sTNFR1 and outcome measures of infarct size and LV-dysfunction. Individuals with the highest levels of sTNFR1 ended up with the largest infarcts and developed a more pronounced LV-dysfunction, which are both markers of a poor prognosis. Similar but weaker and non-significant trends were found for 24 hour levels of sTNFR2. However, the relative increases in sTNFR1 and sTNFR2 over 24 hours were both significantly correlated with infarct size and LV-dysfunction. Notably, plasma soluble FAS and FAS ligand did not consistently correlate with any of the outcome measures.

Several experimental studies have demonstrated the role of TNF signaling in myocardial cell apoptosis leading to IR-injury, LV dysfunction and congestive heart failure [28]. However, data from animal models show conflicting results. In one study, TNF overexpression in mice promotes the development of post myocardial infarction left ventricular remodelling and contractile dysfunction [29], whereas other studies demonstrates the protective role of TNF signaling on myocardial cell apoptosis and on reducing infarct size [30], [31]. While high doses of exogenous TNF renders both ischemic pre- and postconditioning less efficient, pre-ischemic neutralization of TNF reduces infarct size in rabbits and local delivery of sTNFR1 gene reduces infarct size following IR-injury in rats [32]–[34]. A cardioprotective mechanism seems to be conveyed by the TNFR2 receptor when binding to TNF, especially if ligand levels are low [35]. The conflicting results also found in studies on rats further highlight the complexity of the anti- versus proapoptotic properties of TNF signaling [36], [37]. The discrepancies found between different animal models might have several possible explanations. For example, the degree of TNF overexpression varies between animal models and might result in differences in TNFR1 versus TNFR2 activation, resulting in divergent effects on apoptosis. Also, some studies use cardiomyocyte-restricted overexpression of TNF, whereas others overexpress TNF in several cell types (including endothelial cells) possibly leading to different effects on apoptosis during IR-injury. The present study was not designed to evaluate whether TNF signaling exerts anti- or proapoptotic properties, but rather to study correlations between soluble markers of apoptosis and measures of infarct size.

In the present study, we choose to measure sTNFRs instead of TNF in plasma. Data indicate that sTNFRs correlate well with circulating TNF, which due to short half-life in plasma is a less reliable marker [38], [39]. To our knowledge, this is the first study in a pure STEMI population to show the positive association between sTNFR1 in plasma and the extent of the final infarct size. However, these results are in line with previous clinical studies on acute myocardial infarction demonstrating the role of sTNFR1 in predicting mortality and new-onset heart failure [15], [16].

In a previous study on acute myocardial infarction patients, sFAS and sFASL showed no correlation with infarct size, as measured by plasma levels of creatine phosphokinase and of myosin light chain type I [14]. However, sFAS did correlate with hemodynamic measures of heart failure. In our study, including a homogenous STEMI population, sFAS and sFASL did not show any consistent correlations with infarct size, LV-dysfunction or measures of remodeling. However, this does not exclude a role of the FAS/FASL pathway in IR-injury of the myocardium. The time points for analysis of sFAS and sFASL in the present study (at baseline and 24 hours) might not be the optimal in reflecting activation of the FAS/FASL pathway.

Experimental data have demonstrated that intracellular MMP-2 activation in response to oxidative stress might be an important mechanism of triggering cardiomyocyte apoptosis [11]. TNF has been found to provoke cardiomyocyte apoptosis and cardiac remodeling through activation of both intrinsic and extrinsic cell death pathways [40]. In a transgenic mice model with cardiac-restricted overexpression of TNF, there was a significant increase in total MMP activity during the early phase of LV-remodelling [41]. In a previous substudy of the F.I.R.E. trial, we showed a strong positive correlation between plasma levels of MMP-2 and infarct size and LV dysfunction [10]. In the present study, we find a strong positive correlation between plasma MMP-2 levels and all the measured soluble markers of apoptosis. This further strengthens the role of apoptosis in IR-injury.

Left ventricular remodeling was assessed by calculating changes in endsystolic and enddiastolic left ventricular volume indices (dESVI and dEDVI) from day 5 to 4 months after STEMI. None of the soluble markers of apoptosis showed significant correlation with these measures of remodeling. The lack of association between sTNFRs and remodeling, despite correlation with infarct size and left ventricular dysfunction (LVEF), have several possible explanations. The process of remodeling, occurring over a longer period of time, might involve pathways not dependent of TNF signaling or might not be determined by the early increase in TNFR activation following STEMI. Furthermore, in this homogenous STEMI population of patients arriving at the PCI lab within 6 hours of symptom onset and receiving state of the art treatment both in the acute phase and during follow up, infarct sizes were overall rather small and the process of remodeling might have been inhibited to a large degree.

The present study was designed and sized to analyze the effect of FX-06 on soluble markers of apoptosis. No significant differences or trends to differences were found between the treatment groups. Also, the F.I.R.E-study had an overall negative result with no effect of FX-06 on primary or secondary outcomes [18]. Thus, we think it is well justified to analyze all study participants together as one group in relation to outcome measures.

The present study has some limitations. First, there were no prior studies available on soluble markers of apoptosis and cardiac MR measurements to use for power calculations. Therefore, we calculated power based on previous data of biomarkers (MMPs, TIMPs, and TNFR1s), suggesting a sample size of 42 individuals would be adequate. The same power calculation was used in a previously published substudy of the F.I.R.E. trial, where we could detect differences in infarct size and LV-dysfunction in relation to biomarker levels [10]. Nevertheless, we cannot exclude the possibility that the size of the substudy population was too small to detect minor differences in cardiac MR measures, especially since the biomarkers had a non-normal distribution with values within a rather wide range. Secondly, study participants were randomly assigned to receive FX-06 or placebo as adjunctive therapy. Since FX-06 did not have an impact on primary or secondary outcomes in the F.I.R.E trial and did not affect levels of biomarkers in our substudy, we decided to use all patients as one group for the correlation studies. However, we cannot totally exclude the possibility that some minor effects of FX-06 influence the associations between biomarkers and measures of infarct size, LV-dysfunction and remodeling. Thirdly, the increase of circulating soluble TNF receptors is most likely not a cause, but rather a consequence, of an enhanced apoptosis, where apoptotic cells release soluble receptors into the circulation. Furthermore, levels of circulating soluble TNF receptors may not just reflect apoptosis of myocardial cells, but other cell types may also undergo apoptosis during the ischemia-reperfusion phase of STEMI. In addition, soluble TNFRs can also be generated as a result of release from cells of exosome-like vesicles independent of apoptosis [42].

To summarize, in a homogenous STEMI population we find that 24 hour levels of sTNFR1 as well as the relative changes in sTNFR1 and sTNFR2 from 0 to 24 hours are associated with myocardial infarct size as well as left ventricular dysfunction at follow-up. This indicates that apoptosis might be a determinant of the extent of IR-injury. The relative importance of apoptosis versus necrosis during IR-injury needs to be further investigated.
